# Oral Versus Gastrointestinal Mucosal Immune Niches in Homeostasis and Allostasis

**DOI:** 10.3389/fimmu.2021.705206

**Published:** 2021-07-05

**Authors:** Lina J. Suárez, Silie Arboleda, Nikola Angelov, Roger M. Arce

**Affiliations:** ^1^ Departamento de Ciencias Básicas y Medicina Oral, Universidad Nacional de Colombia, Bogotá, Colombia; ^2^ Department of Periodontics and Dental Hygiene, School of Dentistry, University of Texas Health Science Center at Houston, Houston, TX, United States

**Keywords:** mouth mucosa, intestinal mucosa, gastrointestinal tract, immune system, homeostasis, symbiosis, dysbiosis, allostasis

## Abstract

Different body systems (epidermis, respiratory tract, cornea, oral cavity, and gastrointestinal tract) are in continuous direct contact with innocuous and/or potentially harmful external agents, exhibiting dynamic and highly selective interaction throughout the epithelia, which function as both a physical and chemical protective barrier. Resident immune cells in the epithelia are constantly challenged and must distinguish among antigens that must be either tolerated or those to which a response must be mounted for. When such a decision begins to take place in lymphoid foci and/or mucosa-associated lymphoid tissues, the epithelia network of immune surveillance actively dominates both oral and gastrointestinal compartments, which are thought to operate in the same immune continuum. However, anatomical variations clearly differentiate immune processes in both the mouth and gastrointestinal tract that demonstrate a wide array of independent immune responses. From single vs. multiple epithelia cell layers, widespread cell-to-cell junction types, microbial-associated recognition receptors, dendritic cell function as well as related signaling, the objective of this review is to specifically contrast the current knowledge of oral versus gut immune niches in the context of epithelia/lymphoid foci/MALT local immunity and systemic output. Related differences in 1) anatomy 2) cell-to-cell communication 3) antigen capture/processing/presentation 4) signaling in regulatory vs. proinflammatory responses and 5) systemic output consequences and its relations to disease pathogenesis are discussed.

## Introduction

The digestive system is a portal of entry for microorganisms we live and have evolved to coexist within a delicate symbiotic and homeostatic balance that is required for several constitutive processes in the body. Of particular importance, the digestive system is fully covered by mucosal membranes in charge of food breakdown, nutrient absorption, and waste evacuation. As such, the immune system in humans has gone through a long process of self-directed learning of “supervised tolerance” to achieve an ideal mutualistic state with microbial guests. In doing so, the digestive system has specialized its functions by adapting its anatomical features and compartmentalizing immunity with a wide array of different microbial communities along the whole digestive tract. The overall objective of this review is to contrast the current knowledge of oral versus gut immune niches in the context of mucosal immunology and systemic output within the wide spectrum of the digestive system.

## Anatomy of Epithelia-MALT in Oral and GI Tracts

**Figure 1:**
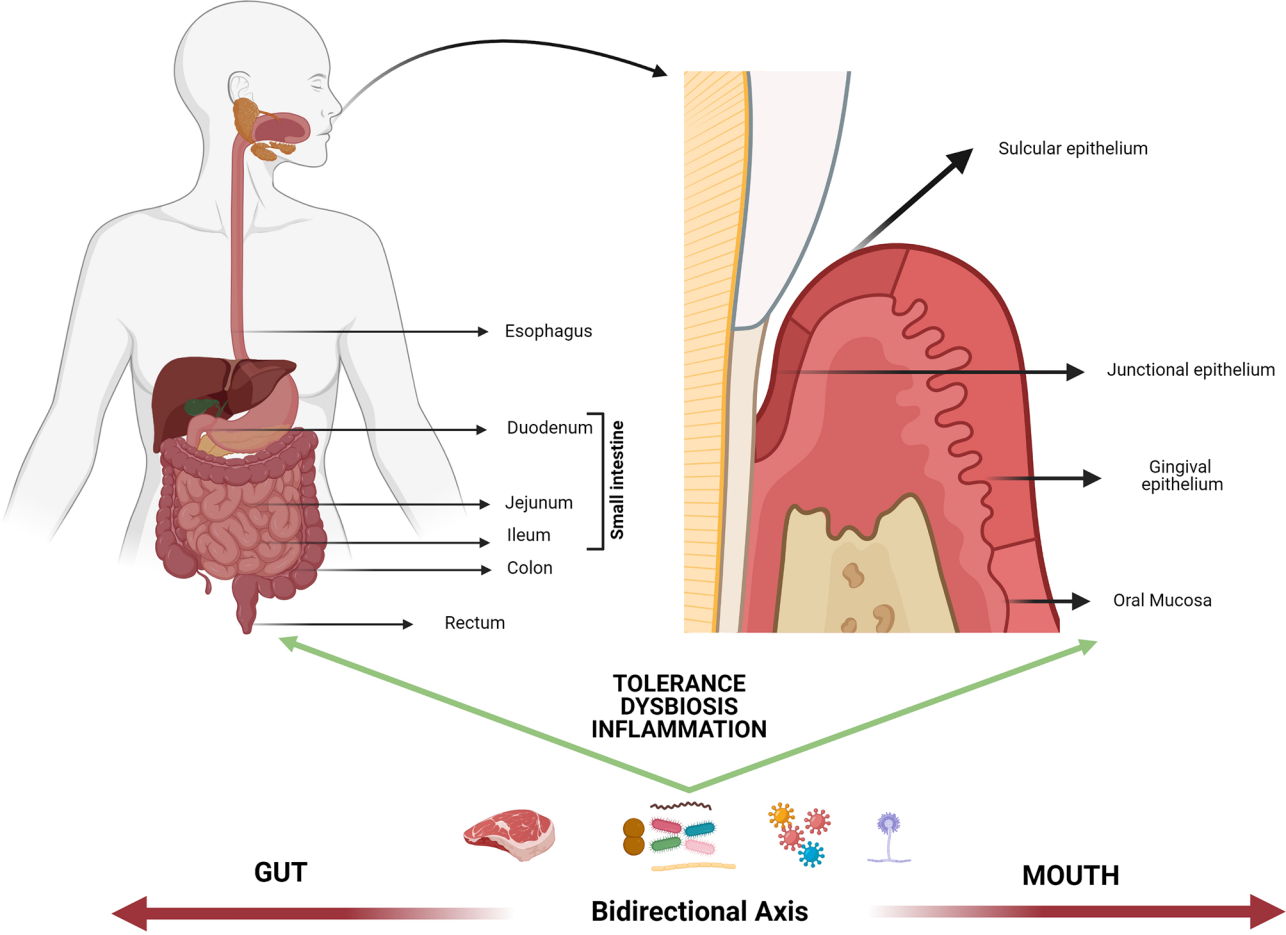
Mouth-gut axis. The plausible interrelation between the oral cavity and the rest of the gastrointestinal system could be mainly mediated by the response to potentially harmful self and/or foreign antigens (i.e., food, allergens, microbiome, trauma), extending beyond the control of tolerance mechanisms. Such mechanisms operate differently based on epithelia diversity of the mouth-gut axis and the direct interaction between the oral/intestinal microbiota and the host immune response, nonetheless they are thought to operate in the same immune continuum. The dysbiosis of microbial communities at both levels as well as the atopobiosis of microorganisms from the oral cavity to the intestine is seen today as a determining factor in the course of multiple inflammatory diseases.

The idea of a bidirectional axis between the mouth and the gastrointestinal tract (GI) is not recent. In fact, possible relationships between the occurrence of pathologies of infectious origin in the oral cavity and inflammatory bowel disorders have been explored for decades (1). Both the oral tract and the GI tract deal with the external environment because of their vital epithelial barrier function in the digestive system, and such function depends on the interrelation between the host and the microorganisms to regulate either homeostatic balance or pathological instability. Despite such similarities, there are certain anatomical peculiarities that allow the understanding of important differences in oral epithelia vs. intestinal epithelium responses to the environment (**Figure 1**).

### Gut Epithelium Barrier

The intestinal epithelium is the second most extensive physical barrier in the human body, just second to the skin (2). As a barrier, its function is to selectively allow the absorption of nutrients, prevent pathogen invasion, prevent loss of water and electrolytes, and allow the exit of waste (3). In addition, the intestinal epithelium is currently recognized as the central axis of mucosal immunity as it is estimated that the gut houses up to 70% of the body’s lymphocyte population, making it the largest immune organ in humans (4). The structural organization of the intestine makes the immune response at the epithelial level different from immunity at the systemic level; such responses are grouped under the name of mucosal immunity. One of the most important characteristics of mucosal immunity is the presence of an inherent lymphoid tissue known as mucosa-associated lymphoid tissue (MALT), which in turn is covered by follicle-associated epithelium (FAE) (5). Although MALT are not unique structures of the intestine, as they are also found in the nasopharynx (NALT) and bronchial tissues (BALT) (6), intestine MALT are fundamental in responding to external antigens.

**Figure 2:**
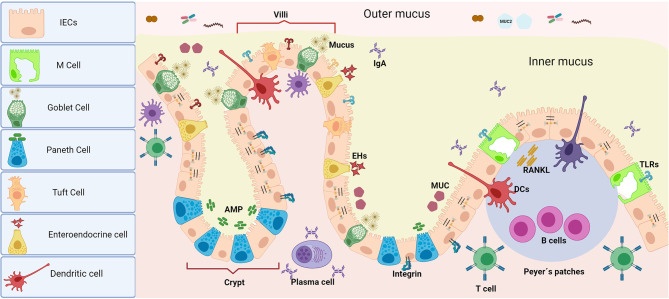
Intestinal epithelium barrier. Unlike oral tissues, the presence of MALT is one of the most important characteristics of mucosal immunity at the intestinal epithelia. The presence of multiple cell types with diverse functions assembled in the intestinal epithelium makes it a highly efficient chemical and physical barrier. In addition to secreting antimicrobial peptides, IECs or absorptive enterocytes control antigen persistence on the surface by epithelial shedding. Paneth cells (which are secretory by nature) are the main source of antimicrobial peptides, which gives them a decisive role in the maintenance of gut-microbiome homeostasis as well as procuring highly efficient turnover of the epithelium. Goblet cells (which are modified epithelial cells) secrete mucins in charge of lubrication and mucus protection, as well as present antigens to CD103+ DCs, complementing the function of M cells. Together, both Goblet and M cells carry out immunosurveillance and transport antigens from the intestinal lumen (from macromolecules to micromolecules) to either activate tolerance or induce an immune response along with specific resident DCs in Peyer’s patches. Tuft cells mainly act as chemosensors, but they are also recognized as immune effector cells with immunomodulatory potential as they have the ability to produce cytokines (including IL25) and play an important role in the expansion of innate lymphoid cells (ILC2). Finally, enteroendocrine cells are responsible for the secretion of enterohormones which are in charge of the body’s response to food as well as modulating physiological events inside and outside of the intestine (i.e., glucose tolerance modulation). The function of the barrier against microbial invasion is completed with a close control of the paracellular permeability given by intercellular junctions of different types, whose structural protein complexes are decisive in their function.

The intestinal epithelium is composed of IECs, which form highly organized structures (villi) and among these, other specific cells such as Paneth cells, Goblet cells, tuft cells, enteroendocrine cells, and M cells are embedded within. Paneth cells protect stem cells by means of antimicrobial peptides (AMP) release (e.g., alpha-defensins, lysozyme C, phospholipases, lectin type C and 3-gamma regenerative islet derivatives -RegIII-) (7). Goblet cells secrete mucins that lubricate and protect the intestinal surface of epithelial cells and also participate in antigen presentation, together with M cells (8). Tuft cells are in charge of chemo-sensing and enteroendocrine cells are hormone-secreting cells (9) (**Figure 2**).

The M or microfold cells are strategically located close to FAE areas. M cells are named for their irregular morphology in which the basement plasma membrane invaginates forming folds/pockets where these immunocompetent cells are housed (5). The epithelium of FAE, compared to the IECs, overexpresses more than 2 times multiple genes related to trafficking through the membrane, host defense and transcriptional regulation, especially ubiquitin D, tumor necrosis factor receptor superfamily 12a, and transmembrane 4 superfamily 4. This specific marker expression pattern differentiates M cells phenotype from IECs and is associated with their specific functions (10). M cells make up 10% of FAE cells and are specialized in taking antigens. M cell markers such as CCL9, Sgne-1, GP2, β1-integrin, PrP, dectin-1, claudin-4, and CD155 have been reported; these usually function as receptors for the different sensing particles (5, 11). M cells depend on RANKL for their differentiation, which is selectively expressed in subepithelial stromal cells in the domes of Peyer’s patches (12). The high interaction of M cells with luminal antigens is due to a reduction in electrostatic repulsion that increases their interaction given through the fucose residues of a glycoprotein coat in M cells (11). M cells also appear to be able to discriminate bacterial antigens and maintain the integrity of the barrier function of the intestinal epithelium through TLR2 (13).

In addition to the presence of multiple phenotypically and functionally different epithelial cells at the level of the intestinal barrier, protection against microbial invasion is also prevented by a close control of paracellular permeability provided by intercellular junctions of different types. Tight junctions, adherent junctions, desmosomes, and gap junctions are lateral structures involved in cell-to-cell adhesion and intracellular signaling (14). The permeability of the epithelium varies throughout the intestinal tract and the composition and abundance of different components of the tight junctions is decisive for their function. Tight junctions are composed of different transmembrane multiprotein complexes that are located in the apical part of the IECs; preventing the passage of large molecules and lipids while allowing the diffusion of ions, water and small compounds through their interaction with actin from the cytoskeleton; aided by adherent junctions. This is known as the apical junction complex. Desmosomes and gap junctions are located below the apical junction complex, and their function is to mediate intercellular adhesion and communication between adjacent epithelial cells (15). Tight junctions function is controlled by signaling molecules such as protein kinase C, mitogen-activated protein kinases, myosin light chain kinase, and Rho GTPases. Intestinal bacteria or food can differentially activate signaling pathways by changing the expression and distribution of tight junctions proteins, thus regulating the function of the intestinal barrier (16). The inter-relationship between the tight junctions and the underlying connective tissue in the lamina propria occurs through the integrins of the basolateral membrane of the epithelium that are attached to the extracellular matrix (14).

The function of the tight junctions is also complemented by that of the adherent functions, which express the proteins E-cadherin, N-cadherin, A-catenin and B-catenin. Such proteins mediate the migration speed of crypts to villus, suppression of proliferation, induction of apoptosis in crypts as well as the rate of differentiation of absorptive cells (17). TLR2 can also mediate intestinal epithelial resistance through redistribution of tight junctions proteins such as zonula occludens-1 (ZO-1) and occludins, thus altering the interaction with microorganisms and metabolites (18). Due to the location of the tight junctions (at the junction between the apical and basolateral plasma membranes), they can also regulate epithelial polarity through paracellular and transcellular pathways that allow the movement of substances to and from the lumen with concomitant energy savings (15). The function of cells and their intercellular junctions is also complemented through the production of an intestinal microclimate, which consists of an undisturbed water layer, the glycocalyx, as well as the mucus layer in which mucins play a fundamental role.

Mucus is mostly secreted by Goblet cells, viscous in consistency and enriched in mucin glycoproteins that form large polymer networks. Mucus secretion is regulated by the host upon detection of intestinal microorganisms or their metabolites (e.g., short chain fatty acids (SCFA) or Th2 cytokines) (19). Goblet cells also participate as luminal antigen presenting cells to CD103+ dendritic cells (DCs), which promote the development of regulatory T cells (Tregs) (20). The organization of mucus is different in all tracts of the intestine. Mucus in the large intestine presents as an external thick layer with abundant bacteria which is not very adherent and an internal “sterile” layer. This mechanism helps trapping bacteria, thereby increasing exposure to defensins and lysozymes (21) as well as separating microorganisms from the epithelium while conferring protection from digestive enzymes. The microbiota residing in the outer gut layers can promote the growth of pathogenic strains but also stimulate biochemical pathways that preserve the structure and function of the intestine. For example, Mucin 2 (MUC2) specifically protects the epithelium from inflammation and thus from multiple disease exacerbation (3). Microorganisms in the outer mucus layer use MUC2 as an energy source, which in turn can lead to mucin-degrading bacteria expanding in the microbiome, thus increasing the degradation of internal mucus (22). MUC2 as well as other components of mucus (CLCA1, FCGBP, AGR2, ZG16, and TFF3) are secreted by Goblet cells that are considered not only as gate-keeping cells but also antigen presenting cells (APCs). MUC2 is a gel-forming mucin, but there are other transmembrane like MUC3, MUC12, and MUC17 that form the glycocalyx (glycolipid and glycoprotein network); they help a two-way communication between structural cells and cells of the immune system, as exemplified in the secretion of IL10. If bacteria can reach the inner mucus layer, they would encounter the compound glycocalyx before reaching tight junctions (23).

The lamina propria underlying the intricate epithelial barrier houses multiple cells, not only DCs but also gut-associated lymphoid tissue (GALT), which includes Peyer’s patches, lymphocytes, and intraepithelial lymphocytes (24). Thus, the lamina propria participates not only in innate but also in acquired immunity through multiple effector responses (secretion of cytokines, IgA, chemokines, proteases, and hormones) mediated by the enteric nervous system, which also regulates intestinal propulsive motility (21).

The stomach and its mucosal layer are also part of the gastrointestinal system; however, unlike the intestine, the gastric mucosa plays a limited immune function as there is no associate-MALT tissues. Current evidence suggests that the gastric mucosa immunity functions in a layer-by-layer progressive mode through innate and adaptive immunity, while maintaining microbiome balance and homeostasis. When pathogens invade the gastric mucosa, both epithelial cells and innate immune cells begin to defend *via* innate immunity; immune cells are rather recruited *via* chemokine signaling and tissue infiltration (25).

### Oral Epithelial Barrier

**Figure 3:**
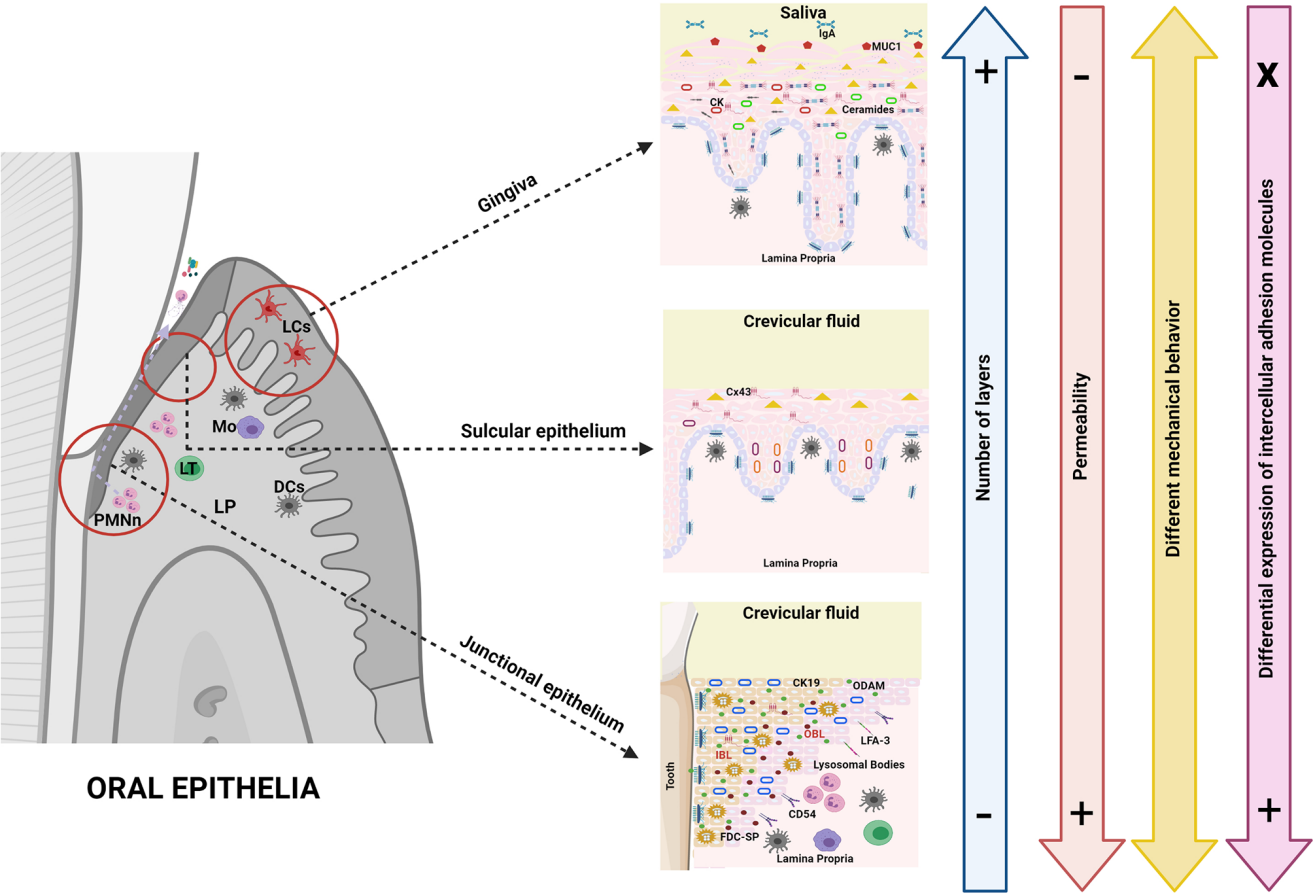
Oral epithelial barriers. Oral epithelia, unlike intestinal epithelia, are multilayered barriers which per se makes antigenic response highly diverse. This fact constitutes an important part of immune tolerance since it could prevent the activation of the response by the simple fact that antigens are not easily detected. The cells that conform the epithelial barriers in the oral cavity are of a similar nature to each other, thus the variations rather occur in complexity, level of differentiation and number of layers, as well as in the expression of conformational proteins of the intercellular junctions (all of the above determining greater or lesser permeability). In the oral cavity, chemosensor, hormone or mucus-producing cells are absent; rather, epithelial cells in oral barriers have different expression patterns of cytokeratins (even in non-keratinized tissues) that are important determinants of the mechanical behavior together with intercellular junctions. The cytokeratin/junction complex can lead to protein expression in the presence of inflammation, ergo modulating the response to different antigens. However, the role of tight junctions in the oral epithelia seems to be less decisive than in the GI tract. The expression of intracellular lipids (ceramides) is also different in oral epithelia; for example, cells of the junctional epithelium show a high production of AMP as well as expression of adhesion molecules that allow the transmigration of immune defense cells in the subgingival environment. Another major difference with the GI epithelia is the absence of MALT which is partly replaced in its function by the oral lymphoid foci. In fact, dendritic cells cannot sense antigens by extending their dendrites through cell junctions in multilayer oral epithelia. Finally, as in the GI tract, the expression of pattern recognition receptors (i.e., TLRs) in oral epithelial cells is highly dynamic, as such expression is inducible and differential against different microorganisms and/or antigens.

Similar to the GI tract, the oral tract is a microenvironment in constant contact with pathogens of all kinds. As in the GI tract, the oral mucosa is a barrier with the external environment and must sense any stimuli to ensure balance of the system (26). The oral mucosa serves as a physical barrier (due to the structural cells and their intercellular junctions in charge of paracellular permeability) as well as chemical barrier, but unlike the intestinal epithelium, oral epithelia are multilayered. Epithelia at the periodontal level include the oral mucosa, the oral epithelium (gingiva), the sulcular epithelium and the junctional epithelium, all of them with different characteristics that are decisive in their function both as a barrier and in their response to the interaction with the oral microbiome (**Figure 3**).

The junctional epithelium, despite being considered stratified epithelia, has just 2 functionally recognized layers: one basal and one suprabasal (27). Junctional epithelium is the tissue with the highest turnover of all oral epithelia (4-6 days) but with very limited differentiation. Junctional epithelium contains specific proteins and differentiation markers such as odontogenic ameloblast associated protein, follicular dendritic cell secreted protein, and cytokeratin 19 (a consistent marker of junctional epithelium) (28), in addition to filaggrin and transglutaminase (29). Lysosomal bodies can be found in large numbers in junctional epithelial cells (27). Unlike the oral epithelium, the sulcular epithelium does not contain a granular stratum but contains a greater number of layers with more differentiated cells than the junctional epithelium with slower proliferation (6-12 days) (29).

Keratinized epithelia (e.g., gingiva and hard palate) have, in addition to keratinocytes, a cornified envelope made up of different types of keratins. Such keratins are composed of extracellular proteins that provide mechanical resistance as well intracellular lipids (ceramides) that regulate permeability (30). In the stratum corneum, there are evenly spaced lamellae and within those spaces there are other disorganized lamellae and electrodense material (membrane lining granules), while in non-keratinized barriers there is a single broad lamella at the periphery of cells. Cholesterol is the main lipid in all epithelia, and ceramides are also part of nonkeratinized epithelia, although in lesser amounts. The buccal stratum corneum is very similar to the stratum corneum of the epidermis in its lipid content and its permeability, but in the gingiva, there is a small portion of phospholipids, much more abundant in non-keratinized tissues and also characterized by low amounts of ceramide 2 as well as abundance of glucosylceramides. In the stratum corneum of the gingiva, the predominant lipids are triglycerides, phospholipids, and glycolipids and their proportions differ with respect to the stratum corneum of the epidermis (31). Therefore, keratins are very important to determine the mechanical behavior of cells and help together with intercellular junctions as support for the barrier in different layers of epithelia (32, 33).

Regarding the underlying layers in the gingiva, the basal layer is composed of mitotic cells that are constantly renewed, which in turn repair the integrity of the barrier (34); whereas in the junctional epithelium, the apical and the basal lamina continues with the basement membrane. Basement membranes are specialized extracellular matrices including collagen types IV and VII, laminin, heparan sulfate proteoglycan, fibronectin, nidogen (entactin), and the proteoglycan perlecan that interpose between connective tissues and the epithelium, endothelium, muscle fibers, and the nervous system. These, in addition to compartmentalizing, participate in selective permeability and molecular screening, cell polarization, migration, adhesion, and healing (27).

As in the IECs, keratinocytes of the oral gingival epithelium are held together by tight junctions and adherent junctions while their attachment to the basement membrane is held by hemidesmosomes (26) these junctions confer paracellular permeability to the oral mucosa, by differential expression of proteins in a similar way to the intestine. There, both desmosomes and adherent junctions function as anchoring/adhesion structures and the expression of conformational proteins varies according to the type of epithelium. For example, there is a high expression of desmoglein 1 and 2 in the cell-cell contacts in the superficial layers in gingival/sulcular epithelium, but not in junctional epithelium. E-Cadherin, a main transmembrane protein of the adherent junctions can be found in the spinous layer of the oral and in the sulcular epithelium but not in the junctional epithelium (35). Thus, it seems that the junctional epithelium has few desmosomes with desmoglein 3 as the main component where adherent junctions are absent.

Connexin 43 (the main protein associated with gap junctions) is related to the activation of calcium hemichannels permeable to small molecules such as metabolites, ions, and intracellular signaling molecules (e.g., ATP, ADP, cAMP, amino acids, small peptides, glucose, inositol triphosphate, cyclic nucleotides, and oligonucleotides) (36). Connexin 43 can be found in the spinous layer of the oral and sulcular epithelium, as well as in some parts of the junctional epithelium, whereas claudin 1 and occludins can be found in the superficial layers of the gingiva. In both oral and sulcular (sulcus/pocket) epithelium, the expression of E-cadherin and involucrin (a key marker of differentiation in stratified epithelia) is decreased in the presence of inflammation, which is associated with marked alterations of actin filaments, indicating advanced damage to the epithelium structure and loss of barrier function (35). As such, all of these proteins support the barrier/antibacterial function properties at the oral/sulcular levels, but not at the level of the junctional epithelium (very few gap junctions can be found in the JE). Nonetheless, the cells of the junctional epithelium are excellent producers of defensins that can be stored in the wide intercellular spaces (37).

Paracellular permeability is based more on membrane coating granules (MCGs) than on tight junctions in stratified oral epithelia (38). In cultures of TR146 cells (a continued cell line of human buccal epithelial origin) have shown tight junctions that are not formed and zone occludens that are not seen (39). However, it has been hypothesized that the differential expression of the tight junctions repertoire in the oral cavity may be involved in the scarring process in this tissue. In a murine model, the expression of claudin 1 and occludin was analyzed and it was concluded that there are genes differentially expressed in oral tissues that can contribute to the mechanisms that lead to the expression of scar phenotypes in response to injuries (40). Keratin also connects to actin through adherent junctions and desmosomes forming a mechanical unit, while adherent junctions act as mechanosensors in the transduction of mechanical forces between the plasma membrane and the cytoskeleton of actomyosin. Desmosomes and intermediate filaments provide mechanical stability to maintain the architecture of these tissues constantly exposed to the action of mechanical forces.

As a chemical barrier saliva (given its high content of IgA, mucins, and antimicrobial peptides) plays a very important role, because of the lining of the oral mucosa. In the mouth, the main mucin is MUC1, which is found in the superficial layer of the oral epithelium and will bind to salivary proteins to form salivary films (41). Noteworthy, it has been determined that MUC1, like MUC4, can also play a role in signal transduction in the immune system. For example, MUC1 through its binding to the B-catenin of the adherent junctions and MUC4 (intramembrane ligand for a receptor tyrosine kinase -ErbB2/HER2/Neu-) activate phosphorylation and initiate corresponding signaling, acting as an important sensor mechanism in response to invasion or damage of the epithelium (42). In the oral mucosa, the membrane-anchored mucin film (MAM) is a mixed film of salivary and epithelial components, and the epithelial cells underlying this protein layer have ridges/folds in the cell membrane called microplicae (MPL), typical of the epithelial cells that are covered by mucus (e.g., esophageal mucosa). MPL vary according to the epithelium of the oral cavity in its morphology: straight, parallel, curved and branched MPL, and honeycomb. Non-keratinized epithelial cells have surfaces with parallel or branching MPL, while keratinized epithelial cells have honeycomb appearing MPL. The exact function of MPL in the oral mucosa is not known but it is thought that they provide mechanical support, maintain the mucus layer, increase the contact surface of the cell with the external environment. This in turn maximizes the absorbance of metabolic products through the external membrane and with the glycocalyx, protects from the entry of bacteria, forming the barrier complex of the oral mucosa with highly glycosylated proteins active in bacterial aggregation. They have also been linked to drug absorption (43).

At the structural level, it is also important to report the expression of intercellular adhesion molecules (ICAM) at the junctional epithelium level. For example, the expression of the carcino-embryonic Ag-related cell adhesion molecule 1 (CEACAM1 mediates cell cohesion) is high in junctional epithelium, which is important during antigen recognition since CEACAM1 functions as a surface receptor for different bacteria, interacting with infiltrating PMNs or T cells and leading to structural alterations to facilitate the immune response (44). Similarly, the expression of intercellular adhesion molecule-1 (ICAM-1 or CD54) and lymphocyte function antigen-3 (LFA-3) is high in junctional epithelium, which supports its important role in inflammatory reactions at this level (27). In sum, the modulation of epithelial barriers at the periodontal level is widely related (although it is not the only factor) to its interaction with microorganisms, which could contribute to the initiation and progression of diseases in the oral tract.

## Epithelia/MALT Interactions and the Fate of the Immune Response in the Underlying Lamina Propria

### Effector Responses in Intestinal and Oral Mucosa

MALT were described more than 40 years ago as organized clusters of B cells and large tissue lymphoid aggregates in which traffic originates to the effector sites in the mucosa (45). MALT has the main function of protecting the host from pathogens but without breaking the integrity of the barrier. For this reason, it is crucial that its activation occurs against antigens that are potentially dangerous while tolerance is exhibited against non-pathogens. MALT must also be activated against dietary antigens and those whose breakdown is related to the occurrence of inflammatory problems.

With the exception of plaque-induced gingivitis, overt inflammation of the oral mucosa is not common due to the highly efficient control mechanisms such as the epithelia which is in constant contact with “invaders” of all kinds (microbial, food, allergens, trauma). Conversely, specific sites of induction of immune responses in the intestine are abundant as they are found embedded in the epithelium, such as the Peyer’s patches, mesenteric, and colon lymphoid nodes (46), housing B and M cells (also present in the nasopharynx) (47) which sense/pass antigens on to professional APCs (follicular DCs, macrophage, and B cells) and additional effector sites, like the intraepithelial lymphocytes in the villi over the lamina propria (48). In fact, there is no MALT in the periodontal tissues, so it is thought that the role of DCs is crucial to process antigens, maturate, migrate to the basal lamina, and present to T cells in structures called oral lymphoid foci. Therefore, the intestinal epithelium has clearly differentiated response-inducing sites while these are absent or at least not characterized in the oral epithelia (49). It is considered that these responses for the oral epithelium rather take place in oropharynx and NALT (50).

Intranasal immunization experiments have led to the understanding that the oral cavity also connects to the cranial and nasal-associated lymphoid tissues (CONALT), which have been proposed to be part of the cervical lymph nodes (CLNs) (51), and play an important role in the induction of immune responses (52). CONALT include the facial lymphoid nodules, parotid, and submaxillary gland (superficial cervical lymphoid nodules), as well as the deep cervical lymph nodules lying dorsal to the brachial plexus in the neck musculature. The lymphoid tissue of Waldeyer’s pharyngeal ring, including the adenoids (the unpaired nasopharyngeal tonsil) and the paired palatine tonsils are also part of the CLNs/CONALT in humans. CONALT help the spread of activated lymphocytes so local responses can be activated. This is facilitated by the expression of homing and addressin receptors that mediate the binding of lymphocytes to high endothelial venules of CONALT, whose expression is differential according to the migration site. For example, peripheral node addressin and L-selectin mediate lymphocyte trafficking to CONALT, addressin cell adhesion molecule-1 (MAdCAM-1) and α4β7 to cervical nodules and VCAM-1 mediate trafficking to lymphoid nodules in parotid gland. Other authors have called this Cranial-oral and nasal-associated lymph nodes CONALNs, or just cervical lymph nodules given the absence of sensor M cells or organized lymphoid structures and the fundamental role of regional lymphoid nodules in the induction of immune responses (53). In sum, the induction of mucosal immunity at distant sites by intranasal immunization proves the existence of a common mucosal immune system (52).

Oral lymphoid foci are expressed under inflammatory pathological conditions in periodontal tissues, and are foci of T, B, and DCs infiltration. They contain Langerhans cells (LCs), Tγδ cells in the epithelium and double-positive maturing CD1a + CD83 + LCs in the lamina propria just under the basement lamina, which could suggest the local presentation of antigens at this level, in addition to the participation of the CONALNs in tolerance induction (49, 54).

### Receptor Engagement in Oral and GI Tracts

Innate immune sensing represents the most ancient form of self/non-self-discrimination (55). For this, evolution has developed a wide array of cellular receptors that recognize more diverse viral, bacterial, fungal, and protozoan surface components, as well as endogenous molecules arising from either constitutive host cell functions or tissue damage coming from trauma or inflammation (56). These innate immune receptors are quite abundant in host cells with many pleiotropic associated pathways, so dissecting each specific agonist/receptor-mediated responses in both the oral and gut tracts is out of focus for this review. Instead, the most important and general pathogen substrate/to/receptor mechanisms in the context of oral and GI innate immunity are reviewed.

#### Toll-Like Receptors (TLRs)

TLRs are a family of transmembrane proteins that have a primary role in pathogen recognition and innate immunity initiation (57). TLRs receptors bind to several microbial components or end-products known as microbial-associated molecular patterns (MAMPs), which include peptidoglycans, lipoteichoic acid, flagellin, double-stranded viral RNA, unmethylated bacterial DNA and lipopolysaccharide (LPS), among others. Currently, 10 human TLRs have been identified, including extracellular as well as intracellular receptors (58). After binding and recognition, TLRs are able to trigger an array of signaling pathways that ultimately activate downstream molecules such as nuclear factor kB (NFkB) and interferon regulatory factor 3 (IRF3) (59), which in turn mediate the expression of several proinflammatory cytokines as demonstrated in several tissues. TLRs are highly involved in responding to inflammatory processes in the presence or absence of infections and are thought to be a critical component of the innate immune response. Their flexibility in recognizing epitopes is facilitated by both physical/structural features, as well as interactions with additional innate immune receptors, soluble molecules, and subcellular trafficking mechanisms (56). In addition to TLRs expression in cells of the immune system such as macrophages and DCs, TLRs are also present in non-immune cells such as epithelial cells, keratinocytes and oral/GI mucosae, making TLRs remarkably important (60).

Oral epithelium acts as the first barrier against invaders of the oral cavity. As such, epithelial sentinel function is of key importance in maintaining tolerance/homeostasis or initiating an immune response. mRNA of all 10 TLRs has been detected in oral epithelial cells, but the actual expression and cellular localization of TLR proteins varies per anatomical location and is inducible under different situations (58). For example, oral epithelial cells do not show increased production of proinflammatory cytokines in response to bacterial components (61) but are rather capable of upregulating TLR and NOD receptors (62). These mechanisms suggest epithelial cells are capable of 1) preventing tissue destruction caused by excessive inflammatory reactions by means of tolerance (63) and 2) initiating and orchestrating an appropriate immune reaction when required. As such, TLRs contribute to the homeostatic relationship between bacteria and the host symbiosis, as previously shown in an *in vivo* model (64).

As the GI tract is heavily colonized by trillions of microorganisms, it is imperative to maintain structural/functional homeostasis during nutrient absorption, commensal/pathogen control and immune regulation crosstalk. As such, TLRs have important antimicrobial functions in preventing excessive responses towards commensal microorganisms as well as activating a strong immune response when appropriate (65). TLR1–TLR9 are expressed in different cell types in the gut, including IECs, immune cells and non-immune parenchymal/stromal cells (66, 67). In particular, human IECs have shown mechanistic capabilities of tolerance and immune regulation *in vitro* similar to oral epithelial cells (68). TLRs are also key regulators of oral tolerance/sensitization in the GI tract. In addition to the commensals and pathogens residing in the gut, food products can often be contaminated by bacteria and/or fungi, so it is plausible that contaminating organisms can also shape oral tolerance to contaminated foods *via* TLRs (69).

TLR signaling is facilitated by the intracellular Toll/IL 1 receptor (TIR) domain (sharing structural homology with the IL1 receptor). The domain itself contains TIR-containing cytoplasmic adaptor molecules (Myeloid differentiation antigen 88 -MyD88-, TRIF) that initiate signaling after dimerization by recruiting additional signaling molecules that ultimately activate the expression of numerous immune response genes through activation of AP-1, NFkB and other transcription factors. MyD88 is an example of a cytoplasmic adaptor protein that drives NFkB translocation into the nucleus and consequently the production of proinflammatory molecules such as prostaglandin E2, leukotriene A4, tumor necrosis factor-alpha (TNFα), IL1β and several chemokines (CXCL8/IL-8, CCL2, CCL3 and CCL5) (56, 70).

#### NOD-Like Receptors (NLR) and the Inflammasome

Leucine-rich repeat-containing receptors (NLRs) consist of about 20 related family members of cytosolic receptors that mostly recognize intracellular ligands. NLRs can be classified into molecules that contain either a CARD (Caspase recruitment domains, or caspase activation and recruitment domain) or a Pyrin motif (71). Nucleotide-Binding Oligomerization Domain Receptors (NOD) CARD proteins mediate NFκB activation, whereas Pyrin molecules (e.g., NALP3) regulate IL1β and IL18 production. NOD receptors recognize peptidoglycan in gram positive bacteria and muramyl dipeptides in gram negative bacteria (58). These receptors are also thought to play a role as antibacterial factors.

Oral epithelial cells express NOD *in vivo* and *in vitro*, playing an essential role in mucosal innate immunity (63). NOD expression is linked with B-defensin production in epithelial cells, which makes them ideal “antibacterial” receptors. In fact, it has been shown that TLR/NOD synergism in oral epithelial cells leads to antimicrobial peptide production instead of proinflammatory cytokine production, reinforcing the theory of “measured” responses to oral bacteria (72, 73).

NLRs also encompass a large number of innate immune sensors and receptors in the GI tract. Similar to oral epithelial cells, several NLRs have been found to restrain, rather than activate, immune signaling and subsequent proinflammatory cytokine production. Noteworthy, while some NLR family members, such as NOD1 and NOD2, can cause activation of these pathways in response to stimuli in the gut, select NLRs, such as NLRX1, NLRC3, and NLRP12, act as negative regulators of inflammatory pathways, adding to the complexity of NLR biology (74).

NLRs binding and downstream signaling can elicit an inflammatory reaction by means of cytokines, chemokines and antimicrobial peptides production. Some products can be proinflammatory (IL6, IL8, TNFα) while others can display immunoregulatory or antimicrobial properties (interferon gamma, IFNγ and human β-defensin-1, hBD-1). Importantly, one major function of NLR proteins involves the modulation of inflammatory signaling pathways, including NFκB and MAPK (58). Also, another well-defined function of some NLRs (NLRP1, NLRP3, NLRC4, and NLRP6) is the activation of a multi-protein complex, known as the inflammasome.

The inflammasome is a large multiprotein complex that recognizes a wide array of microbial, stress and danger signals, triggering the maturation of proinflammatory cytokines, including IL1β and IL18 and promoting innate immunity (75). NLRs regulate caspase-1 activation as the initial step of inflammasome formation. Even though caspases are cysteine proteases that are mostly known to regulate cellular death *via* apoptosis, they also facilitate proinflammatory cellular processes. As such, caspases can be categorized as either proinflammatory or proapoptotic (76, 77).

#### Protease-Activated Receptors (PAR)

PAR receptors are a family of four G protein-coupled receptors that sense proteases. Proteases, or proteolytic enzymes, are majorly involved in all signal transduction events in both homeostasis and disease, as they can be derived from circulation (coagulation factors), inflammation (mast cells/neutrophils) or from other multiple sources (epithelial cells, neurons, bacteria, fungi); as such, proteases can act at the cellular surface level by generating or destroying receptor agonists as well as activating/inactivating surface receptors, thereby making the PAR system of vital contribution for a “backup” signal transduction events (78). Much is known about PARs functions, however understanding the specific role of proteases in physiology and pathophysiology remains largely unknown.

PAR family members PAR1 and PAR2 are highly expressed in the oral periodontium. These receptors participate in periodontal tissue metabolism by regulating inflammation and repair processes through activation of endogenous factors and/or bacterial enzymes (79). Interestingly, the keystone pathogen *Porphyromonas gingivalis* (*P. gingivalis*) is known to produce gingipain enzymes which specifically cleave PARs as part of its immune evasion mechanisms (80). Also, the abnormal activation of PAR2, also known as coagulation factor II (thrombin) receptor-like 1 (F2RL1) drives several pathophysiologic processes in the oral cavity, including oral cancers, as PAR2 promotes oral squamous cell carcinoma growth/progression *in vitro* (81).

PARs are highly expressed in the GI tract while PAR2 is the best studied. The most probable reason for the high expression of these receptors is the fact that the GI tract is continuously exposed to a wide variety of housekeeping or bacterial proteases, like digestive enzymes or proteinases, respectively. PARs play important roles in enterocyte function, intestinal ion transport, GI motility and exocrine secretion, and act as effectors of intestinal inflammatory disorders (82). Like most GPCRs, PARs couple to multiple signaling pathways and can thereby regulate many cellular functions such as cellular proliferation, migration, secretion, adhesion, and transcription.

### Antigen Capture/Presentation

DCs are considered to be the most important actors of antigen presentation at the cellular level in both the oral and GI barriers. DCs must not only attain defense against the invasion of pathogens but must also limit the immune response even after the encounter with the antigen. Therefore, the benefits that can be obtained from the encounter with commensals or from the intake of nutrients are not altered by an uncontrolled activation of the host immune system (49). DCs are specialized APCs which control a spectrum of innate and adaptive responses (83). DCs are a heterogeneous population of cells, distinguishable by surface, intracellular phenotypic markers, immunological function, and anatomic distribution (84). However, DCs have recently been defined as cells of the hematopoietic system on their own (85). Dcs are found throughout the lymphatic system but also in non-lymphoid tissues such as skin and mucosal surfaces (85), which are the most common sites of entrance for microbial pathogens (84).

DCs have been classified into five main cell types: conventional DCs type 1 and 2, monocyte-derived DCs, LCs and plasmacytoid DCs. The establishment of transcriptomic similarities between mouse and human DCs types lead to the creation of this classification (86). Each subtype resides in a different part of the body and has specific roles in the immune response (85). In most tissues, DCs are present in an immunologically immature state as immune sentinels poised to respond (87). These DCs lack the necessary accessory signals for T cell activation. Nevertheless, they are well equipped with pattern-recognition receptors (PRRs) both on and within many immune cells in the peripheral tissues of both GI and oral tracts (88). DCs capture and process antigens to form MHC–peptide complexes *via* endocytic pathways such as phagocytosis or macropinocytosis (85). DCs are also in constant movement throughout their life cycle (85), because their ability to migrate throughout the body is a critical aspect for the initiation of immunity (87). DCs also play an essential role for the discrimination of antigens, leading to autoimmune disease (89).

DCs are widely distributed residents in the oral epithelium. Myeloid DCs and LCs are the most common phenotypes, expressing CD1a and CD207 (LCs specific lectin Langerin) similarly to their intestinal counterparts. However, oral and GI DCs differ in the expression of costimulatory molecules B7.1 (CD80) and B7.2 (CD86) as well as other myeloid markers such as CD11b (49). The amount and distribution of DCs varies in the oral mucosa depending on the site, with less presence in the gingiva and the sublingual region than in other areas of the mouth (90). Oral DCs express significantly more MHC class I and II and CD40, as well as Fc*γ*RIII/CD16 and Fc*γ*RI/CD64 receptors. They express a high affinity receptor for IgE (FcϵRI) even in non-atopic individuals, indicating high allogeneic stimulation. Therefore, DCs contribute beyond responses to infections in the oral cavity, such as response to allergens, for example (91). Interestingly, it is possible to find alterations in the amounts of DCs of different types in lichen planus (92) and oral carcinomas. Immature DCs (CD1a+) and LCs (CD207+) are significantly decreased in oral submucous fibrosis and oral squamous cell carcinoma, when compared to a normal oral epithelium. Additionally, an increase in plasmacytoid DCs (CD303+) was reported in oral squamous cell carcinoma, indicating possible relationship with the development of these pathologies (93).

Antigen-presenting DCs in the intestinal mucosa mostly remain in an immature state in the presence of commensals but encounter with new, potentially pathogenic antigen can initiate an active immune response. DCs in GI mucosal tissues are somehow instructed by IECs to suppress inflammation and promote tolerance, but DCs that are not “tolerated” are recruited to from Peyer’s patches and circulating blood to the insult site, consequently initiating an inflammatory response. In doing so, the differential response of IECs to both commensals and pathogens controls DCs-mediated responses, while activated T and B cells in Peyer’s patches are marked to return to the intestine given DCs ability to promote on-site antigen presentation through upregulation of integrin 47 and CCR9 (94). Additionally, these Peyer’s patches DCs promote IgA secretion by inducing B cell differentiation (95). Such mechanism has not been reported in the oral cavity although it has been assumed that absence of increased DCs recruitment in the oral cavity could prevent over-activation at the level of the oral mucosa (49). Remarkably, *in vitro* co-culture experiments with oral epithelial cells and DCs in the presence of gram positive and gram-negative bacteria does not lead to DCs maturation, as evidenced by low MHC II, CD80 and CD86 expression with very little IL12 and TNFα when compared to non-activated DCs. Similarly, DCs in co-culture were not able to stimulate allogeneic naive CD4+ to produce INFγ and TNFα, when placed under these conditions or in the presence of Th1, anti-CD3 and anti-CD28 cells. This is thought to be one of the strategies of oral epithelial cells to avoid hyper reaction of the immune system against resident bacteria, through the inherent ability of this epithelial barrier to suppress immune responses (96). The other strategy is related to DCs allowing T cell clones to acquire memory by persisting long-term periods, tolerating self-antigens, or responding rapidly upon re-exposure to noxious antigens (83).

Macrophages are also critical antigen-presenting cells with important roles in both tissue homeostasis and inflammation of the oral and gastrointestinal tracts, as phenotypic M1 (proinflammatory pathogen-killing) and M2 (cell proliferation/tissue repair) polarization in resident macrophages can be found in the gum/gut axis (97). For example, M1 has been associated to chronic periodontitis whereas M2 is associated to health/gingivitis stages (98); nonetheless M2 has also been associated to chronic infections (99). Similarly, the GI tract is one of the most macrophage-dense organs. In a surveillance state, colonic macrophages exhibit a M2-like phenotype supported by CD206+/CD163+ phenotype, IL-10 production, promoting epithelial cell proliferation/regeneration and promotion of Tregs proliferation; whereas several mouse IBS studies show that M1 proinflammatory macrophages dominate the large intestine during experimental colitis (100). In this regard, oral and gut are remarkably similar, however, there are clear imbalances in M1/M2 ratio responses as these are heavily influenced by the immune microenvironment.

### Adaptive Immunity in Oral and Intestinal Immune Niches

Microbial processing and clearance are essential for appropriate immune response and efficient disease resolution. DCs take up pathogens and/or antigens by means of receptor mediated phagocytosis and process these to be presented to other cells of the immune system. Phagocytosis provides an opportunity for DCs to sense the nature of the engulfed “invader” so efficient intracellular routing to specific compartments tailors an appropriate immune response (101). Lysosomal degradation then takes place through multiple mechanisms including the endocytic/phagocytic pathway (102), macroautophagy (103), microautophagy (104) and chaperone-mediated autophagy (105). Interestingly, such pathways can be subjected to bacterial exploitation (106).

Some differences between oral and gut can be described in the context of antigen presentation and immune consequence. For example, the oral mucosa is considered to be very tolerogenic in spite of continuous exposure to a wide array of antigens (commensal or pathogenic) or mechanical trauma (107), as evidenced by the fact that severe inflammatory responses in the oral cavity are relatively rare (49, 108). Furthermore, a distinctive arrange of tolerogenic Dcs compartmentalized in different locations in the oral epithelium that induce regulatory CD4 + T cells has been described, making the oral tract an ideal site for inducing tolerance (109). In the case of food allergies are mostly mediated by a type 2 immune reaction to dietary antigens processed by gut APCs (primarily different types of DCs) leading to the initial priming of T cells and the production of food specific IgE antibodies. Thus, DCs (along with monocytes and macrophages) dictate gut tolerance to allergy by shaping the T cell and subsequent B cell antibody response (110).

How DCs know where to go specifically continues to be a mystery, and to date this is still under investigation (87). Upon activation, DCs switch their behavior from endocytosis towards migration (85). Throughout this journey migrating DCs must adapt their motility skills to reach their target destination, including the capacity to traverse a wide variety of tissue types and across many anatomic barriers, recognize and adhere to specific microvascular endothelial cells, sense and follow chemoattractant signals, and interact with lymphocytes and other immune cells to allow the exchange of critical information regarding the antigens-presenting process (84, 85).

DCs undergo cytoskeletal changes to optimize DCs motility that results in fast migration (85). Expression of the G-protein coupled receptor CCR7 located on the surface of DCs enhances their migratory capacity towards the lymphatic tissues (87). CCL19 and CCL21 are chemokines expressed by peripheral lymphatic endothelial cells that guide DCs to downstream lymph nodes (84). The interaction between the chemokine CCL21 and its receptor CCR7 is crucial for the migration of activated DCs (85). Most chemoattractant signals result in integrin activation, which causes firm adhesion and arrest of the cells leaving the blood vessels *via* diapedesis (85). A variety of cytokines and chemokines are released in response to bacterial products. Granulocyte-macrophage colony-stimulating factor (GM-CSF), TNFα, and IL1 have been described to promote DCs movement and maturation (87). Many molecular processes take place in DCs migration but the most important factor for DCs arrival at the lymph node is the chemokine-mediated guidance (85).

Once into the lymphoid tissues, DCs may complete their maturation, release chemokines that attract B and T lymphocytes, and maintain the viability of recirculating T cells (87). In the words of Ralph Steinman -the discovery of DCs- the maturation process of DCs is a critical link between innate and adaptive T cell-dependent immunity (111). Innate immunity includes rapid reactions to infection and is not specific to a particular pathogen (83). Adaptive immunity is acquired more slowly (days to weeks) and includes highly specific responses for antigens that are sustained long-term to develop improved function upon re-exposure to the antigen (83). Nonetheless, adaptive immune responses can be both immunogenic or tolerogenic (112).

Maturating DCs both activate and expand T helper-cells (Th), controlling many T cell responses (87). These Th cells then exert both inflammatory and regulatory responses (112). For example, DCs induce different types of CD4+ T cells such as Th1, Th2, or Th17 to produce powerful cytokines, which increase resistance to infection (83). The balance between subsets of T cell effector populations has been determined to be important in the quiescence as well as the progressive stages of inflammatory diseases. IL17 is a pro-inflammatory cytokine secreted by CD4+ Th17 cells that stimulates the recruitment of neutrophils and monocytes into inflamed areas (83). The role of Th17 cells is to maintain mucosal barriers and contribute to bacterial clearance at mucosal surfaces (88). In terms of regulation of the inflammatory response in the oral epithelium, there is ample evidence demonstrating the key role that Th17/Treg balance can play in the pathology of oral diseases, such as periodontitis (as in the proportion of Th17 and its products in gingival tissues can explain the severity of periodontitis) (113).

The role of the dysbiotic changes that guide Th17 subpopulations differentiation at the local level also has an impact at the systemic level, that could support the relationship between inflammatory periodontitis and other systemic diseases and could aid in diagnostic/prognostic testing (114). Recent studies have shown an association between IL17 and many inflammatory and autoimmune diseases such as systemic lupus erythematosus, sclerosis multiple, asthma and inflammatory bowel disease (IBD) (112). Alternatively, tolerogenic DCs can eliminate or block T cells, resulting in resolution of ongoing immune responses and prevention of autoimmune responses, which despite having been reported as widely prevalent in oral mucosa, seem to be related to non-pathologic responses and even represent non-recognized physiological functions as the immune removal of debris (115, 116). Alternatively, tolerogenic DCs can eliminate or block T cells, resulting in resolution of ongoing immune responses and prevention of autoimmunity (115).

The regulation of inflammation at all levels relies to a large extent on the cells that express the FOXP3 marker, which acts as a master regulator of the inflammation regulation pathways and the development of Tregs. FOXP3+ cells are generated in the thymus or induced in peripheral tissues, so their functions are different. Accordingly, thymic cells will be selected by their own antigens to control systemic immunity while peripheral cells are induced by antigens in the tissue and are related to local suppression (117). Current evidence suggests that Tregs in oral mucosa reach sites by migration and not by induction *in situ* (118). In addition, a high presence of FOXP3+ cells (Tregs) can be phenotypically different from those of lymphoid nodules or spleen, based on the differential expression of CD103+, CTLA-4, CD44+ and Neuropilin-1. The oral mucosa presents large amounts of different phenotypes of CD4+ cells with relatively known functions, unlike the intestinal mucosa where CD8+ cells predominate (119). Therefore, it could be speculated that the CD4+FOXP3+CD25+ phenotype in the mouth leads to protection against possible autoimmune reactions or against commensal organisms (120). In fact, it has been described in mice that these FOXP3 cells in oral mucosa are mainly in the lamina propria which enables such capability (119).

The process of inducing tolerance in the gut is not entirely clear but sensing of antigens by the immune system is thought to be involved. Immune responses vary if the antigen is captured from the lumen, through the gap junctions of goblet cells, versus if antigens are captured and presented by various APCs in the lamina propria (121–123). Most intestinal Tregs cells are Tr1, Th3 and CD4+CD25 + and are believed to interact with DCs while TrE CD8+ cells recognize antigens presented by epithelial cells (124). This DCs-T cell interaction is crucial in the induction of tolerance, so if a naive T cell recognizes an antigen presented by an immature dendritic then it will not differentiate into an effector cell but rather into a Tregs. In addition to gap junctions, MUC2 of Goblet cells promotes tolerogenic properties in DCs that induce pTregs, including TGFβ production and retinaldehyde dehydrogenase expression. Glycans associated with MUC2 determine anti-inflammatory properties in DCs by assembling a galectin-3-Dectin-1-FcγRIIB complex that activates β-catenin that in turn inhibits the activation of NFkB by altering the expression of pro-inflammatory (not tolerogenic) cytokines (125).

Under inflammatory conditions, Tregs that conventionally produce IL10, TGFβ and IL35, can also produce pro-inflammatory cytokines such as INFγ and IL17A (based on their expression of RORγt) (126). It is not very clear whether they retain their suppressive capacity or contribute to inflammation, as this depends on environmental signals, local presence of proinflammatory cytokines, and metabolites. Such phenomenon makes targeting Tregs therapeutically difficult (127). Tregs can also upregulate the expression of pro-inflammatory cytokines (IL-17A and IL-22) dependent on IL-2 stimulation in a murine model of infection by candida albicans (128), although this is still controversial (129). Furthermore, short chain fatty acids correlate with an increase in the frequency of Foxp3 +, IL-17A +, and Foxp3 + IL-17A + double positive (Treg17) in lingual tissue and oral draining lymph nodes after depletion of resident bacteria by the antibiotics use, highlighting the role of the resident microflora during mucosal infections (130).

In addition to the regulation of inflammation by the induction of Tregs, DCs (like most cells) release extracellular vesicles that are an important part of immune regulatory mechanisms, participating not only in antigen presentation but also in cell-to-cell communication (131). Extracellular vesicles are small nanometric molecules that are divided into three groups according to their biogenesis: apoptotic bodies, microvesicles, and exosomes (132). All cells, including DCs can use this mechanism to carry out exchanges of proteins, lipids, nucleic acids, signaling molecules, and modulating responses (133). For example, during an infection with adherent-invasive *Escherichia Coli* (associated with Crohn’s disease), exosomes secreted by infected cells can have an impact on the innate immune responses of surrounding cells to infection at the intestinal level, which can in turn be amplified by the same exosomes and contribute to associated diseases. In fact, exosomes have been suggested as diagnostic markers and therapeutic targets to both diagnose and treat intestinal inflammation (134). Interestingly, exosomes isolated from infected DCs have the ability to reprogram immune cells responsible for experimental alveolar bone loss *in vivo* (135).

## Systemic Output and Its Relations to Pathology

### Microbiome and Symbiosis

Microbiome is an ecological community of commensal, symbiotic, and pathogenic microorganisms that share our body space and have been determinants of health and disease (136). The composition of the microbiome varies across body places and among individuals (137). Compiled data from the MetaHit and the Human Microbiome Project identified 2,172 species isolated from humans, classified into 12 different phyla, from which 386 are strictly anaerobic and therefore will usually be found in mucosal regions such as the oral cavity and the GI tract (138). The collection of microorganisms including bacteria, archaea and eukarya that live in the GI tract is named the “gut microbiota” and has coevolved with the host to form a mutually beneficial relationship (138). The gut microbiota is the largest microbiome in our body with more than 50 different phyla and 500 bacterial species (139). However, a healthy human gut microbiota is dominated by three primary phyla: *Firmicutes* (30-50%), *Bacteroidetes* (20-40%), and *Actinobacteria* (1-10%) (139). Strict anaerobes such as *Bacteroides, Eubacterium, Bifidobacterium, Fusobacterium, Atopobium*, and *Peptostreptococcus* are major constituents of the gut microbiota, whereas facultative anaerobes including *Lactobacilli*, *Enterococci*, *Streptococci*, and *Enterobacteriaceae* are present in less proportion (139). The composition of the microbiota varies along the gut*. Bacteroidetes* and *Actinobacteria* represent more than 90% of bacterial phyla in the colon but only 50% in the small intestine, which contains around 40% *Firmicutes* species (139). The majority of gut bacteria are non-pathogenic and cohabit with the enterocytes in microenvironments within the intestine reaching a healthy homeostatic equilibrium known as symbiosis (140, 141). The gut microbiota offers several benefits to the host through a range of physiological functions including nutrient and drug metabolism, maintenance of structural integrity of the gut mucosal barrier, immunomodulation, and protection against pathogens (139).

On the other hand, the oral cavity harboring more than 770 prokaryotic species (according to the expanded Human Oral Microbiome Database) houses the second largest and diverse microbiome (142). Different habitats in the mouth (e.g., teeth, gingival sulcus, tongue, cheek, and hard and soft palate) form a diverse ecological system that allows the growth of different microbial communities, including bacteria, fungi and viruses creating the “oral microbiota” (143). Bacteria are the most common microorganisms found in the oral microbiome. Although several bacterial phyla have been described to date, the oral cavity is dominated by *Firmicutes* (36.7%), *Bacteroidetes* (17.1%), *Proteobacteria* (17.1%), *Actinobacteria* (11.6%), *Spirochaetes* (7.9%), and *Fusobacteria* (5.2%) (142). Under normal conditions, these sets of microorganisms live harmonically in communities structurally and functionally organized called biofilms. These bacteria cohabit in the oral biofilms with synergistic and antagonistic interactions that contribute to ecological stability (144). Salivary components are the major nutritional source for microbial growth. IgA, lactoferrin, lactoperoxidase, lysozyme, statherin, and histatins are required for the development of a balanced microbiome. Saliva has antimicrobial properties due to presence of certain components including nitrite and hypothiocyanite that contribute to the state of equilibrium of the microbiome (144).

Homeostasis of both oral and gut microbiomes is characterized by diverse and dynamic microbial communities. However, the oral microbiome is more diverse and dynamic as compared to the gut microbiome (145, 146). The biodiversity of this ecosystem improves its ability to resist environmental disturbances, and dynamic microbiomes are more selective of colonizing bacterial members (146). The microbiota influences the induction, training, and function of the mucosal immune system. Conversely, the immune system has evolved to allow the maintenance of a symbiotic relationship of the host with this complex ecosystem (139, 147). The immune system is directed to reinforce the immunity barrier and thereby their own containment (147). One of the most important strategies used by the host to maintain this homeostatic relationship with the oral and gut microbiota is to minimize contact between microorganisms and the epithelial cells surface, limiting tissue inflammation and microbial translocation (147). In the GI tract, house of the largest density of commensals, several mechanisms benefit a tolerogenic immune response. In the small intestine, this objective is achieved by the action of PRRs, antimicrobial peptides, IgA, CD103+ DCs and regulatory T cells as well as cytokines including IL10, IL33, and TGFβ. In the large intestine a thick continuous mucus layer favors this goal. These structural and immunological defense mechanisms have been termed as the “mucosa firewall” (148). The active sampling of commensal, pathogens and antigens is mediated by three types of immunosensory cells (i.e., enterocytes, M cells, and intestinal DCs) (149). The gut microbiota interacts with the host cells through a well-regulated immune system that involves TLRs and NLRs. The capacity of immune cells to discriminate commensal from pathogenic bacteria is mediated partially by these receptors (149). It has been shown that intestinal epithelial integrity can be enhanced through the activation of TLRs, which results in proliferation, restitution, and protection of IECs against apoptosis. Commensal bacteria could also induce the expression of NOD2, which plays an important role in regulating the intestinal mucosal homeostasis and suppressing colonization with pathogenic bacteria (149). Commensals also control pathogenic flora through the competition for nutrients and production of antimicrobial peptides that affect the survival and virulence of pathogens (147). As a result of the interactions between the microbiome and the immune system, microbial attacks are effectively controlled by the host response, thereby maintaining a symbiotic state. Although all these processes in oral and GI mucosa induce a protective response that prevents the host from developing diseases in most cases, there is potential for these mechanisms to be disturbed either by overgrowth of microorganisms or by changes in the local host response as a result a dysbiotic state (150). Lastly, evidence from studies in germ-free animals has shown the important role of commensal bacteria on intestinal homeostasis. Germ-free animals are more predisposed to infections, have reduced vascularity and slower renewal of epithelial cells. Anatomically, such animals present longer intestinal villi, associated with crypt atrophy and smaller Peyer’s patches with fewer intraepithelial lymphocytes. In addition, the mucosa and muscle wall thickness were decreased in these mice (139). Gut microbiota promotes the preservation of the integrity of the intestinal epithelial barrier and the promotion of epithelial repair after injury (139).

### Dysbiosis and Anatomic Alterations

Dysbiosis is defined as a condition in which the normal microbiome population structure is disturbed (150). The ability of a microbe within the microbiota to cause or exacerbate diseases varies. Most microbes can shift from one relationship to another based on the state of activation of the host, infection, and localization (147). Diseases, some aspects of the lifestyle (e.g., smoking, diet, oral hygiene), genetic variations, antibiotic treatments, the activity of salivary proteins, salivary flow rates, innate/adaptive immune factors, as well as pathogens have been associated with dysbiosis (137, 144). Disruption of the balance of the bacterial ecosystems in the human microbiome has been associated with a plethora of inflammatory diseases and infections including some GI disorders and periodontitis (143).

Periodontitis is a common disease in the oral cavity. The pathogenesis and development of periodontitis involves a complex gene-environment interaction model rather than the earlier proposed model caused by one or two single factors (151). There is no doubt that periodontitis is initiated by bacteria. However, the triad of microorganisms commonly named as “red complex” (*P. gingivalis-, Treponema denticola -T. denticola-*, and *Tannerella forsythia -T. forsythia-*), are not the only ones involved in the etiology (152). Evolving advances in the periodontal research field have indicated dysbiotic microbial community may be responsible for eliciting progressive inflammation and alveolar bone loss (152). Indeed, the host inflammatory response and other modifying and predisposing factors determine the clinical outcome of periodontitis (151). It is now well established that environmental factors (e.g., smoking, diet, and stress), immunoregulatory defects associated with polymorphisms or mutations, systemic diseases, aging, epigenetic modifications, and the presence of keystone pathogens may disrupt the ecosystem transforming a symbiotic microbiota into a dysbiotic one and promote the initiation of periodontitis in a susceptible host (146, 151, 153). The transition from periodontal health to disease is associated with a remarkable shift in the composition of subgingival communities (154). A symbiotic community is composed mainly of facultative bacterial genera including *Streptococci* and *Actinomyces* while a dysbiotic community is composed of anaerobic genera from the phyla *Firmicutes, Proteobacteria, Spirochaetes* and *Bacteroidetes* (155). Abusleme et al. have reported that the change occurred in community structure rather than shifts in membership. That means that most taxa seen in periodontitis were commonly seen in health although in a small number and in low proportion. Conversely, most health-associated taxa were commonly seen in periodontitis. Therefore, the ecological shifts from symbiosis to dysbiosis are a consequence of the increment of dominant species, rather than the disappearance of health-associated species. It was also reported that periodontitis is associated with enhanced uniformity thus contrasts with other disorders of the GI, in which dysbiosis is associated with a decrease in bacterial diversity (155).

In early stages of gingivitis, neutrophils are the first cells to arrive chemotactically at the inflammatory infiltrate through intercellular spaces in the junctional epithelium (156). The presence of pathogens into the subgingival crevice or pocket is followed by chemotactic cytokines secretion by epithelial cells, making the neutrophils release proteolytic enzymes disrupting the epithelial barrier. This gingival epithelial disruption allows pathogens and their products to infiltrate into the lamina propria, triggering the release of pro-inflammatory cytokines and inducing tissue breakdown and bone loss. When antigens enter the lamina propria, they can be taken up by DCs thus activating TNFα, INFγ and IL-13, which increase inflammation. In addition, the contribution of periodontopathogens and their virulence factors to barrier disruption have been studied (156). The gram negative, black pigmented bacterium *P. gingivalis* is the most widely studied periodontal pathogen; and is considered as a keystone in the development of periodontitis (152). A variety of virulence factors including LPS, fimbriae, hemagglutinins and gingipains contribute to the pathogenicity of this microorganism (156). Katz et al. have demonstrated that *P. gingivalis* disrupted the epithelial integrity and is able to invade the connective tissue by degrading epithelial cell-cell junctions complexes, thereby allowing the spread of the bacterium (157). Likewise, *T. denticola* degraded the ZO-1 protein, disrupting the epithelial barrier and infiltrating the epithelial layers (156). *A. actinomicetemcomitans* decreases connexin-43 levels and E-cadherin expression in gingival epithelial cells (156). Taken together, these results suggest that beneficial bacteria and their products maintain the gingival epithelial barrier by improving tight junction-related gene expression and by developing a beneficial microenvironment that reduces the viability of barrier-disrupted pathogens (156).

**Figure 4:**
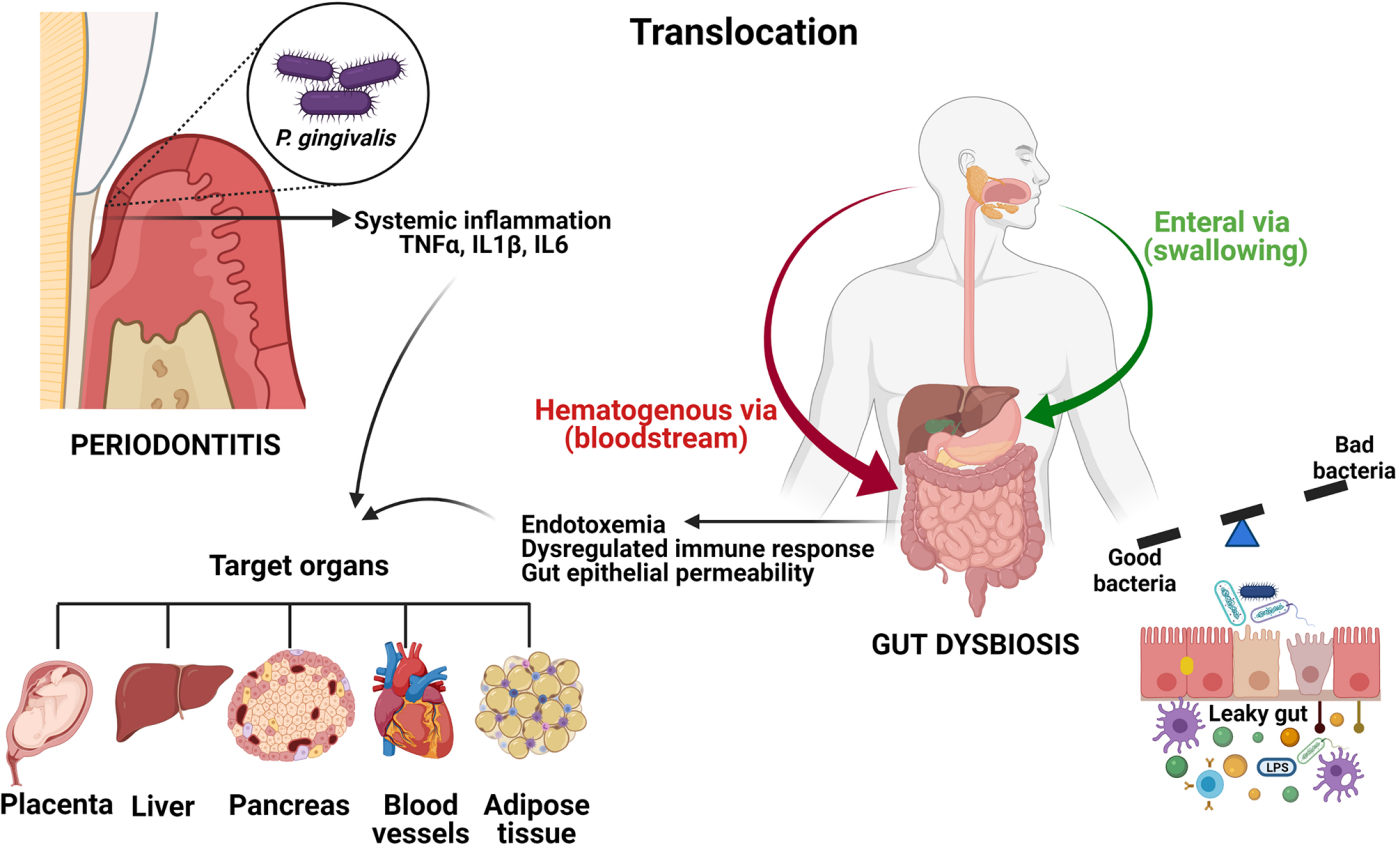
Oral and GI systemic connections. Oral bacteria can translocate to the digestive tract enterally or parenterally. In periodontitis, locally pro-inflammatory mediators including TNFα, IL1β and IL6 can enter the systemic circulation and induce a low-grade systemic inflammation. Disruption of the gut epithelial barrier induced by dysbiosis results in increased gut epithelial permeability, thus creating a leaky gut. This leakage generates endotoxemia, systemic inflammation and a dysregulated immune response. Since some diseases such as type 2 diabetes, non-alcoholic fatty liver disease, obesity and atherosclerosis have been associated with periodontitis, as well as with gut dysbiosis, it is reasonable to assume that swallowing oral bacteria *via* saliva could represent a possible mechanism linking periodontitis and systemic diseases.The figures were created with BioRender.com.

On the other hand, GI permeability regulation is mediated by anti-inflammatory cytokines (microbiome-guided expression), while failure in the permeability of the barrier during GI inflammatory diseases can be attributed to the alteration in the expression of proinflammatory cytokines (158). Gut dysbiosis and other factors may trigger epithelial barrier dysfunction which contributes to the increased intestinal permeability, thus creating a “leaky gut” (159, 160). Other alterations of intestinal protection mechanisms including reduced secretory Ig A and generalized decreases in immunity could further affect the intestinal barrier. This leaky gut allows the entrance of bacteria and their products (MAMPs) from the gut lumen into the host. Once inside the intestinal immune system, these MAMPs interact with innate sensors such as TLRs of intestinal cells and intracellular NLRs, triggering an inflammatory response characterized by increased production of IL1, IL6, IL8 and TNFα (160). As a consequence, cytokine imbalance perpetuates intestinal inflammation, which may trigger an array of autoimmune diseases (143, 161). Moreover, leaky gut allows certain commensal bacteria in gut microbiota to escape the lumen of the gut inducing inflammation and causing systemic tissue damage once translocated into the peripheral circulation. Gut bacteria can be translocated to the liver *via* portal vein. Into the liver, these PAMPs activate hepatocytes and Kupfer cells through TLRs and NLRs such as innate immune responses including cytokine production, leading to hepatic injury (162) (**Figure 4**).

### Systemic Disease Output (Oral vs. GI and Systemic Connections)

The oral and gut microbiomes are anatomically connected as they colonize mucosal surfaces in the digestive system. As a result, mechanical and/or biochemical alterations can affect both in a bidirectional way (145, 150). In fact, oral and gut microbiota may be two of the most important microbiomes affecting overall human health (145).

Although the GI tract is highly effective in preventing colonization of foreign microbes, certain oral bacteria can spread from the oral cavity and be translocated to the digestive tract through both parenteral (hematogenous) and enteral routes (163). Segata et al, reported that an estimated of 1x10^11^ bacterial cells per day flow from the mouth to the stomach, which means that the oral microbiome might considerably contribute to distal digestive tract populations (164). Species of *Bacteroides, Eubacterium, Streptococcus, Prevotella*, and *Veillonella*, among others have been detected in both the oral cavity and stool in more than 45% of subjects in the Human Microbiome Project (164). In addition, saliva influences the microbial growth of the habitats above the stomach because saliva is a buffer and controls the pH, while its high mucin content allows for nutrient availability. Patients with intestinal diseases commonly exhibit abnormal enrichment of common oral bacteria in the luminal contents and the gut mucosal tissues. In fact, bacteria exclusive to the oral cavity, such as *Campylobacter, Porphyromonas, Prevotella, Fusobacterium* and *Actinomyces* can be found in the gut of patients with gastrointestinal diseases (143).

There is ample scientific evidence demonstrating the translocation of periodontal pathogens from the local periodontium to end-organ targets is facilitated by hematogenous systemic dissemination (157). In fact, epidemiological data suggests that periodontitis is associated with an increased risk of a plethora of diseases including type 2 diabetes, atherosclerotic vascular diseases, adverse pregnancy outcomes, obesity, rheumatoid arthritis, and non-alcoholic fatty liver disease (154, 165). Patients with severe periodontitis can swallow 1x10^12^-10^13^ *P. gingivalis* each day (162). Theoretically, if oral bacteria can resist the pH of the stomach, they may potentially reside and colonize the GI tract. *P. gingivalis* is resistant to acid gastric, which permits migration to the colon with subsequent local effects (163). Several studies have focused on investigating if *P. gingivalis* can experimentally modulate the gut microbiome. Studies in mice have shown that *P. gingivalis* can disrupt the gut epithelial integrity through reduced expression of tight junction proteins as well as modifying the microbial composition, both *via* outgrowing in the gut and disseminating *via* endotoxemia (162, 165). Arimatsu et al. reported that oral administration of *P. gingivalis* strain W83 (1x10^9^ CFU/ml twice a week for five weeks) in mice induced a change of bacterial composition in the ileum, accompanied by increases in the levels of plasma endotoxin, insulin resistance, and systemic inflammation. Reduction in the mRNA expression of the tight junction proteins ZO-1 in the ileum was also reported. Authors hypothesized that the endotoxemia likely was not derived from *P. gingivalis* but could be related to disturbances of the gut microbiota caused by swallowed bacteria leading to metabolic disorders (165). Similarly, Nakajima et al. have reported that a single administration of 1x10^9^ CFU/ml *of P. gingivalis* (strain W83) in mice induced dysbiosis of the gut microbiota, with increased *Bacteroidetes*, decreased *Firmicutes*, and increased serum endotoxin levels. The gene expressions of tight junction protein-1 and occludin (involved in intestinal permeability) were also downregulated. Higher amounts of bacterial DNA on the liver of infected mice were also reported. It is important to highlight that changes in intestinal microbiota preceded systemic inflammation, supporting the idea that alterations of the gut microbiota composition by swallowed periodontopathic bacteria may be a causal mechanism linking periodontitis and systemic diseases (162). However, it is worth noting that these experiments used human oral bacteria, which are exogenous to the mouse microbiome, hence limiting direct extrapolations to human pathogenesis. In sum, the periodontal microbiome affecting the gut microbiome requires much more clinical evidence while experimental results provide a new paradigm to understand the role of periodontopathic bacteria in the gut microbial composition and the plausible development of systemic diseases (165).

*Fusobacterium nucleatum (F. nucleatum)* is one of the most abundant microorganisms in the oral cavity, in both healthy and diseased individuals. *F. nucleatum* is a pathobiont that outgrows during oral dysbiosis and may act as a bridging organism, allowing for other keystone bacteria to bind *via* adhesins, thus playing a key role in periodontitis. Compelling evidence shows that *F. nucleatum* is found in the colonic mucosa of patients with IBD and colorectal cancer (143). These findings were followed by studies showing that *F. nucletaum* may play a role in colorectal cancer development, metastasis and disease outcome (166). Adhesion to the gut epithelium is mediated through surface proteins including FadA, Fap2, and RadD. Mechanisms by which *F. nucleatum* is involved in tumor progression include the creation of a proinflammatory microenvironment and TLR4-activated signaling to NFkB. Additionally, *F. nucleatum* may induce immune suppression of intestinal mucosa by affecting the function of immune cells including macrophages, T cells, and natural killer cells. The ability of *F. nucleatum* to induce resistance to chemotherapy in colorectal cancer is mediated *via* TLR4/NFkB pathway-induced autophagy. Since *F. nucleatum* is involved with the development of colorectal cancer, metastasis, and treatment outcome, strategies to selectively target *F. nucleatum* should be further explored in the future (166).

There is growing evidence that intestinal flora plays a key role in human physiology and dysbiosis of the gut microbiota is associated with the pathogenesis of several diseases within and outside the gut (145). Intestinal disorders comprise IBD, irritable bowel syndrome (IBS) and coeliac disease (CD), whereas extraintestinal disorders include asthma, allergy, cardiovascular disease, metabolic syndrome, and obesity. IBD is an idiopathic condition that causes chronic inflammation of the digestive tract and includes Crohn’s disease and ulcerative colitis (143). The dramatic increases in the prevalence of IBD in recent years have made it one of the most studied imbalances between microbes and the immune system. Host genetics are involved in the IBD onset, with genetic factors being more critical for the development of Crohn’s disease than ulcerative colitis. However, environmental factors are unequivocally involved (167). The contribution of the gut microbiota together with a dysregulated immune response is the current accepted disease hypothesis. Several studies have shown that gut dysbiosis in patients with IBD is characterized by a decrease in the bacterial diversity, temporal stability, and cluster separately when compared to healthy controls. Results from animal models in colitis require the presence of intestinal bacteria to initiate inflammation, and an increased mucosal bacterial load is observed in IBD patients. These studies have also identified polymorphisms in genes involved in bacterial recognition and clearance. Noteworthy, inconsistent results have been found in some microbial compositional comparisons. Nevertheless, these studies have generally identified reductions in components of the *Firmicutes* phyla with concurrent increases in *Bacteroidetes* and facultative anaerobes such as *Enterobacteriaceae* (167).

IBS is a common disorder of gut-brain interaction worldwide (168). The gut-brain axis describes the bidirectional interaction between the emotional and cognitive areas of the CNS and the peripheral function of the GI tract (168, 169). Recently, the “brain-gut” axis is referred to as the “brain-gut-microbiota” axis (168). Although the etiology is not fully understood, changes in gut microbiota have been characterized in the different subtypes of disease compared to healthy subjects (170). The distribution of subtypes include IBS-diarrhea, IBS-constipation, and mixed IBS (168). The presence of a specific microbial signature characterizing these diseases is still unknown. However, decreased microbial diversity and the presence of *Clostridiales* species (methane-producing bacteria) associated with severe symptoms have been described (169). In general, epidemiological data suggest that there is a relative abundance of proinflammatory bacterial species including *Enterobacteriaceae* with decreased *Lactobacilli* and *Bifidobacterium*. When IBS patients are compared to healthy individuals there is an increase in *Firmicutes* to *Bacteroidetes* ratio and increased levels of *Streptococci* and *Ruminococcus* species are observed (168). In addition, genetics, diet, GI infections and psychological factors have been also proposed as potential risk factors for the development of IBS (169).

CD is a chronic intestinal inflammatory disorder due to an aberrant immune response to the ingestion of gluten proteins in susceptible subjects (171). Although the etiology is multifactorial this disorder is strongly associated with the expression of the leukocyte antigen DQ2 (170). There is evidence that a notable reduction in gram-positive bacterial populations, as well as increase of total gram-negative bacteria is characteristic of the active phase of the disease, which can contribute to loss of gluten tolerance. These results confirm that structural changes in the composition of the gut microbiota are associated with CD. In addition, lower levels of IgA-coated bacteria have been detected in CD patients compared to healthy controls. These results support the hypothesis that there is an intestinal barrier defect in CD patients, which fails to stabilize the gut microbiota and allows the entry of harmful antigens and pathogens, all triggering an inflammatory response (171).

Similarly to periodontitis, no specific pathogen seems to cause IBD, IBS, and CD (161). Periodontitis and these GI disorders share in common a dysregulated host immune response due to disruption by microbial communities or by changes in the local host immune response (154, 167). Consequently, the failure to control self-directed immune responses triggered by environmental or microbiota-derived antigens in susceptible individuals results in tissue damage.

## Conclusions

Epithelial tissues are crucial in establishing tolerance in multiple human body systems in direct contact with the external environment including the skin, the respiratory system and more crucially with the digestive tract (oral and GI compartments). However, beyond the ability to maintain a “non-responsive” state, epithelia are in charge of both having a symbiotic relationship with the microbiome and maintaining health, as well as inducing the activation of inflammatory processes to control aggressions. Therefore, epithelial-mediated inflammation *via* “sentinel receptors” (TLRs, NLRs, etc.) can be associated with clinical signs of multiple diseases in the presence of the host immune response deregulation and/or microbial dysbiosis. Although the final objective of establishing tolerance in both oral and GI epithelia is ultimately similar, the anatomical considerations here presented as well as the compartmentalization of the immune response make each pathway widely different, albeit traditionally considered part of the same continuum.

The initial host microbiome interaction with the oral epithelia is relatively unknown, while the initial antigen sensing process deciding the fate of the response is better documented in the gastrointestinal epithelia. However, it is clear that the initial antigen sensing in the oral cavity must be a highly regulated process, as this could be hypothesized from the low incidence of oral inflammatory epithelial diseases in humans (with the notable exception of periodontitis). In addition, there is also a gap in the knowledge of how different foods could alter the inflammatory processes and impact the response of the host in metabolic activities at buccal level, as well as what would be the influence of the oral microbiome in the process.

Although clear differences in epithelial function have been presented here, the authors conclusion is that undeniably, in light of the evidence, there is a strong relationship between oral microbiota translocation to the GI tract with a subsequent systemic disease occurrence triggered by the alteration in intestinal permeability. Therefore, such relationship between inflammatory oral diseases mediated by translocating microorganisms is becoming more and more relevant in overall systemic health and makes the oral microbiota a future therapeutic target for the prevention and treatment of multiple systemic diseases.

## Author Contributions

All authors listed have made a substantial, direct, and intellectual contribution to the work, and approved it for publication.

## Funding

The corresponding author (RA) is supported by the Robert Wood Johnson Foundation HAMFDP102806.

## Conflict of Interest

The authors declare that the research was conducted in the absence of any commercial or financial relationships that could be construed as a potential conflict of interest.
